# Enhanced magnetic refrigeration properties in Mn-rich Ni-Mn-Sn ribbons by optimal annealing

**DOI:** 10.1038/srep11010

**Published:** 2015-06-09

**Authors:** Yu Zhang, Linlin Zhang, Qiang Zheng, Xinqi Zheng, Ming Li, Juan Du, Aru Yan

**Affiliations:** 1 Key Laboratory of Magnetic Materials and Devices, Ningbo Institute of Material Technology and Engineering, Chinese Academy of Sciences, 1219 Zhongguan West Road, Ningbo, 315201, People’s Republic of China; 2 School of Materials Science and Engineering, Ningbo University of Technology, Ningbo, 315016, People’s Republic of China; 3 School of Materials Science and Engineering, University of Science & Technology of Beijing, 100083, People’s Republic of China

## Abstract

The influence of annealing time on temperature range of martensitic phase transition (ΔT_A-M_), thermal hysteresis (ΔT_hys_), magnetic hysteresis loss (ΔM_hys_), magnetic entropy change (ΔS_M_) and relative refrigeration capacity (RC) of the Mn-rich Ni_43_Mn_46_Sn_11_ melt spun ribbons have been systematically studied. By optimal annealing, an extremely large ΔS_M_ of 43.2 J.kg^−1^K^−1^ and a maximum RC of 221.0 J.kg^−1^ could be obtained respectively in a field change of 5 T. Both ΔT_A-M_ and ΔT_hys_ decreases after annealing, while ΔM_hys_ and ΔS_M_ first dramatically increase to a maximum then degenerates as increase of annealing time. A large effective cooling capacity (RC_eff_) of 115.4 J.kg^−1^ was achieved in 60 min annealed ribbons, which increased 75% compared with that unannealed ribbons. The evolution of magnetic properties and magnetocaloric effect has been discussed and proved by atomic ordering degree, microstructure and composition analysis.

Magnetic refrigeration based on magnetocaloric effect (MCE) is a potential refrigeration technology with high efficiency, low cost, and environmentally friendly, which is drawing more attention as an alternative to the existing vapor compression refrigeration. Recently, magnetic refrigerants, such as Gd-Si-Ge^1^, La-Fe-Si^2^,^3^, Mn-Fe-P-As^4^ and Ni-Mn-based Heusler alloys^5^ come to be research focus due to their giant MCE caused by first-order phase transition. But from the point of application, it is important for a magnetic refrigeration material not only has a large magnetic entropy change (ΔS_M_) but also a large refrigeration capacity (RC)^6^,^7^,^8^. Amorphous structure is in favor of a large RC while in a compensation for a relative low ΔS_M_^9^,^10^,^11^. Therefore, how to increase RC but keep a large ΔS_M_ at same time is a key issue for a magnetic refrigeration material.

Among them, rare-earth-free Ni-Mn-X (X =Sn, In, Sb) Heusler alloy systems have aroused people’s great interest due to their large inverse MCE^12^,^13^,^14^. Additionally, for Ni-Mn-Sn system, its low cost of raw materials, easy fabrication, low energy cost during fabrication etc. make it having more advantages for applications. While, except for MCE, martensitic transition related other magnetic properties such as magnetic hysteresis loss (ΔM_hys_), cooling capacity (RC) are important factors, which need to be considered before its application^15^,^16^. Even these parameters are intrinsic, but they are more sensitive to microstructure, microcomposition, phase/magnetic ordering degree, which are strongly influenced by fabrication conditions.

Current research mostly focuses on bulk alloys, synthesized by conventional arc melting followed by prolonged high-temperature annealing. More recently reported melt-spinning technique effectively promotes more homogeneous single-phase materials, substantially shortened annealing stage and improved MCE properties^17^,^18^. But both samples prepared by these two methods need to be annealed for a period of time in order to obtain a uniform phase. Because of the longer atom diffusion distance in bulk sample, it needs longer annealing time to obtain homogeneous composition. Ghosh and Aksoy *et al*.^19^,^20^ have also studied the influence of annealing time on magnetocaloric effect of Ni-Mn-Sn bulk samples at 1173 K from 0 h to 24 h and a maximum magnetic entropy change could be found when annealing time is close to 24 h. In our recent research, an extremely large MCE more than 40 J.kg^−1^K^−1^ in a field change of 5 T was achieved in annealed melt-spun ribbon sample compared with bulk sample of 29.5 J.kg^−1^K^−1^^21^. For melt-spun ribbons, short time^17^ about several minutes or longer time^18^ around several hours has been reported separately. It is obvious that the annealing time has great influence on magnetocaloric effect of Ni-Mn-Sn melt spun ribbons. But it is still not very clear which annealing time is optimal. And there are few reports on systematically studying what’s the important factors influence on refrigeration related parameters for this MCE system.

To our knowledge, a maximum magnetic entropy change ΔS_M_ of 33 J.kg^−1^K^−1^ was reported for a Ni-rich alloy of Ni_51.6_Mn_32.9_Sn_15.5_ due to a field change of 5 T, which is almost a record MCE for Ni-Mn-Sn family alloy^5^. In this article, an extremely large ΔS_M_ of 43.2 J.kg^−1^K^−1^ and a high effective RC_eff_ more than 100 J.kg^−1^ were obtained in Mn-rich Ni_43_Mn_46_Sn_11_ ribbons by optimizing annealing time. The influence of annealing time on martensitic transition related behaviors and magnetocaloric effect of Mn-rich Ni_43_Mn_46_Sn_11_ melt spun ribbons was systematically investigated and discussed. The evolution of microstructure, composition, atomic ordering degree has been introduced to explain the trend of magnetic refrigeration related properties on different annealing time.

## Results and discussions

As discussed in the introduction part, it is rare to find an optimal annealing time for melt spun ribbons. In the literatures, either extremely short or long annealing time would miss an optimal annealing condition, which prevented achieving optimal magnetic refrigeration properties. Here, different annealing time from 0 min to 180 mins has been carried out for Mn-rich Ni_43_Mn_46_Sn_11_ melt spun ribbons, and martensitic transition (MT) related magnetic behaviors and MCE has been systematically studied.

### Thermal Magnetization, Temperature Range of Martensitic-Austenitic Transition and Thermal Hysteresis

**Figure 1:**
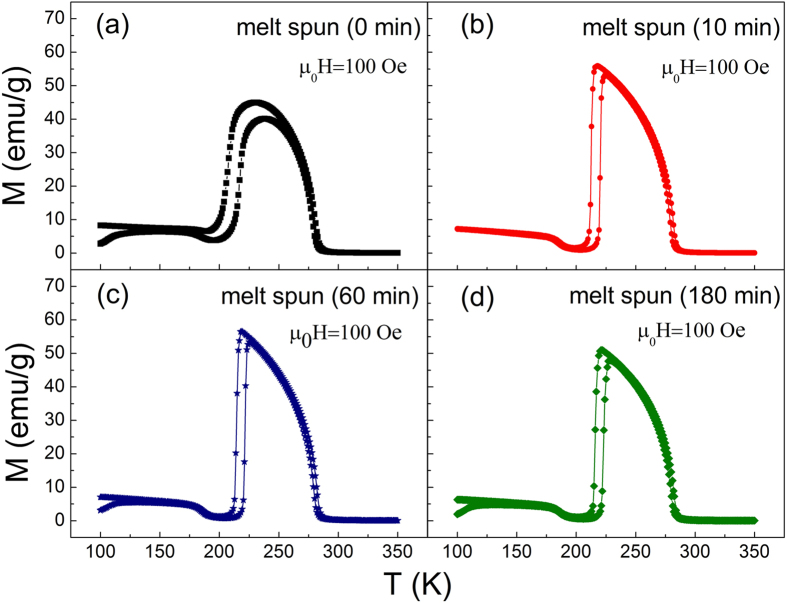
Temperature dependence of magnetization (M-T curves) for ribbons annealed for different annealing time, (**a**) as spun ribbon, (**b**) 10 min, (**c**) 60 min^21^, (**d**) 180 min in the presence of 100Oe field.

The temperature dependence of magnetization during heating and cooling at a rate of 10 K/min for Ni_43_Mn_46_Sn_11_ ribbons annealed at different time in a low magnetic field of 100 Oe was shown in Fig.1. In the thermal magnetization curve, an abrupt change in magnetization around 200 K belongs to martensitic transformation (MT) proved by many previous researches^22^,^23^. The MT in Ni-Mn-X (X = Ga, In, Sn, Sb) alloys takes place from a cubic austenitic phase, showing L2_1_ Heusler crystal structure (space group *Fm3*^-^*m*) and next-nearest neighbor atomic order, to a low-symmetry martensitic structure^13^,^24^. Accordingly, the phase changes from high temperature ferromagnetic (FM) austenitic phase having larger susceptibility, to low temperature martensitic phase, which exhibits small magnetic susceptibility. The temperature range for this transformation ΔT_A-M_ can be calculated using the following formula:


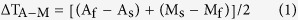


Where A_s_, A_f_, and M_s_, M_f_ are the starting and finishing temperatures of the austenite and martensitic phase, respectively. With the increasing of annealing time, the value of *A*_*s*_ slightly increases as listed in Table 1. Moreover, the temperature range of martensitic-austenitic phase transition (ΔT_A-M_) decreased dramatically from 11 K for unannealed sample to 3 K, 3.5 K and 4 K for 10 min, 60 min and 180 min annealed samples. It’s obvious that the unannealed one has the widest ΔT_A-M_ and correspondingly the thermal hysteresis ΔT_hys_ during this phase transition is largest for the as spun ribbons (10 K), and then reduces to 7 K after annealing as listed in Table 1. The annealing time has no much different effect on ΔT_hys_.

For the as spun ribbon, the composition is inhomogeneous even it has single L2_1_ phase tested by XRD after rapid solidification from the melt, as a result it has larger ΔT_A-M_, which can be ascribed to the following two reasons. One is that the atoms in the unannealed sample (as spun ribbon) are inhomogeneous and disordered. The other reason is that due to the fast cooling rate, the defects and stress are reserved in the as spun ribbons. The decrease of ΔT_A-M_ for the annealed ribbons is due to the homogeneity of the composition and reducing of the defects and stresses. And along with the more homogeneous composition after different time annealing, atomic order degree (AOD) would also increase, which was the reason why *A*_*s*_ has a little bit increase, and which may also contribute to the narrower ΔT_A-M._ At the same time, the magnetic refrigeration performance would also be strongly influenced by annealing, which will be shown and discussed later. However, the composition may be less homogeneous when the annealing time is more than enough, which would have a negative influence on AOD, thus mildly widen the temperature change of phase transformation, and the increased ΔT_A-M_ from 3 K for 10 min sample to 4 K for 180 min annealed sample can further prove it. The change of compositional homogeneity will be proved in the following part by EDS in SEM (see Table 2). The change of atomic ordering degree AOD will be confirmed by the XRD analysis in the following part (see Table 1).

### Isothermal Magnetization and Magnetic Entropy Change ΔS_M_

**Figure 2:**
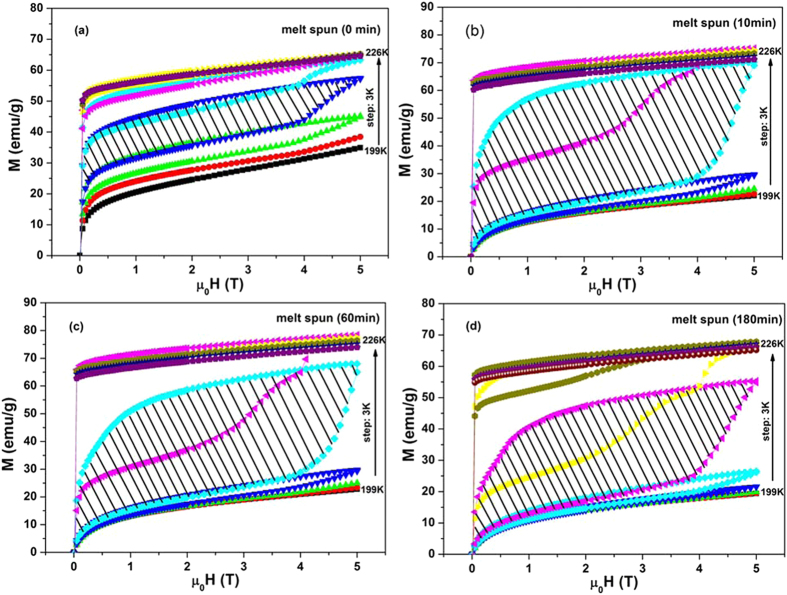
Isothermal magnetization (M–H) for (**a**) as spun ribbon, (**b**) 10 min annealed ribbon, (**c**) 60 min annealed ribbon^21^, (**d**) 180 min annealed ribbon, near martensitic phase transition temperature.

In order to investigate magnetic refrigeration performance, isothermal magnetization (M-H) curves up to 50 kOe near T_M_ for melt-spun ribbons annealed for different time were measured as shown in Fig. 2. From the M-H curves, it is obvious that all the samples show a typical ferromagnetic behavior above 217 K, which presents austenitic phase. And a metamagnetic behavior between 205 K and 217 K was due to a field-induced martensitic transition. Apparently, the increasing manner of magnetization around 211 K for 10 min annealed ribbons is much sharper than all the other most obvious metamagnetic transition curves for the other three samples. Another important difference needs to be mentioned is that the magnetization of austenitic phase M_A_ increased from 65 emu/g for the as melt-spun ribbons to about 75 emu/g for the annealed 10 min and 60 min ribbons, but decreased to 67 emu/g as further increase annealing time to 180 min. Meanwhile, the magnetization of martensitic phase M_M_ decreased from 35 emu/g for unannealed ribbons to around 20 emu/g for 10 min, 60 min and 180 min annealed ribbons.

The change of magnetization both for austenitic phase and martensitic phase is due to different annealing time, which affects the magnetic correlations in the alloy. In the austenite phase, there are two positions for Mn atoms, one is the regular Mn site and another is the Sn site, and both of their magnetic moments order ferromagnetically^19^. After annealing, an increase in the ordering of Mn atomic moments to their respective proper sites happened and as a result, the ferromagnetic correlation increases, which directly leads to an increase in M_A_. While, the antiferromagnetic correlations between Mn-Mn at different positions strengthened after annealing as the structure of these materials transforms from austenitic phase to martensitic phase on cooling, which results in a decrease of M_M_^20^. The deep reason for the change of magnetization can be ascribed to atomic ordering degree, which will be discussed later. Since there is an increase of M_A_ and decrease of M_M_ upon annealing as listed in Table 1, the difference in magnetization between austenitic phase and martensitic phase (ΔM_A-M_ listed in Table 1) was is enlarged, which is favorable to obtain a large magnetic entropy change.

Martensitic transition is a phase transition associated with structure change, which belongs to a first order transition. As mentioned in the first part, a thermal hysteresis ΔT_hys_ is obvious as shown in Fig. 1 and listed in Table 1. Usually, the trend of magnetic hysteresis loss ΔM_hys_ upon annealing is similar with that of ΔT_hys_ for the first order transition. But in our research, it has a different trend in Ni-Mn-Sn alloy. The dashed area in Fig. 2 represents the maximum ΔM_hys_ during phase transition for each sample and it means that the larger the dashed area, the larger the hysteresis loss. The values of the largest ΔM_hys_ for four samples are listed in Table 1, respectively. It first dramatically increases from 57.1 J.kg^−1^ for the as spun ribbons to 185.5 J.kg^−1^ for the 10 min annealed ribbons, and then it gradually decreases to 168.6 J.kg^−1^ for the 60 min annealed sample and 123.3 J.kg^−1^ for 180 min annealed sample. But the annealing time has no much difference on ΔT_hys_. It is obvious that the longer the annealing time, the smaller the ΔM_hys_. The change of ΔM_hys_ upon different annealing time will be discussed in the following part.

**Figure 3:**
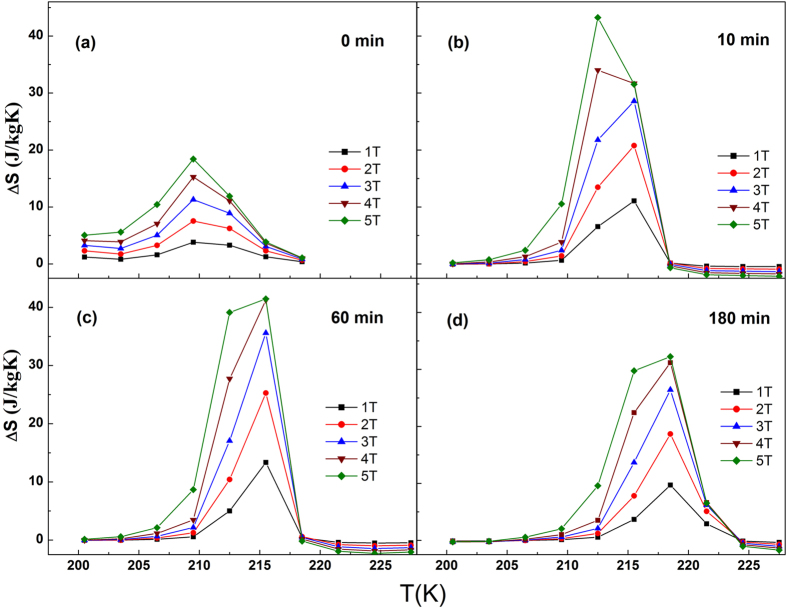
ΔS_M_ as a function of temperature for (**a**) as spun ribbon, (**b**) 10 min, (**c**) 60 min^21^ and (**d**) 180 min annealed ribbon due to 1.0−5.0 T field change.

The ΔS_M_ of all the samples has been estimated using Maxwell’s thermodynamic relation:


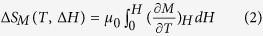


where, T, M, H, and μ_0_ are the temperature, magnetization, magnetic field intensity, and permeability of free space, respectively. The M-H curves used for the calculation of ΔS_M_ are the curves measured during field increasing. The temperature dependences of magnetic entropy change ΔS_M_ for the samples of as spun and annealed ribbons are plotted in Fig. 3. It can be seen that the ΔS_M_ for the annealed samples increase dramatically compare with that of the as spun ribbons as listed in Table 1, which can be explained by the increase of atom order degree AOD by annealing in the following part. A maximum ΔS_M_ of 43.2 J.kg^−1^K^−1^ in a field change of 5 T is obtained in the sample annealed for 10 min. As compared to other samples, the largest value of ΔS_M_ for this sample originates from the sharpest change in magnetization during martensite/austenite structural phase transition, which could be well explained by the increase of atomic ordering degree in the following part. While for the other two annealed samples, the metamagnetic transition is not directly change from martensitic phase to austenitic phase (which can be proved by Fig. 2), but to an intermediate state, therefore the ΔS_M_ is not as large as that of 10 min annealed sample even the difference in magnetization between two phases is similar. On the other hand, ΔS_M_ will decrease with the increasing of annealing time. In our research, it is postulated that the decrease of atomic ordering degree and decrease of compositional homogeneity would result in the depression of ΔS_M_. And, this will be proved in the following section.

### Refrigeration Capacity (RC), Magnetic hysteresis, and Microstructure

For a magnetocaloric effect material, refrigeration capacity (RC) is an important parameter to be evaluated for its potential application, which estimates the total amount of thermal energy that can be transferred to a hot sink from a cold source in one ideal thermodynamic cycle. RC has been calculated using ΔS_M_ as a function of temperature and integrated it within the full width at half maximum (FWHM) of ΔS_M_ of the materials^7^. Upon annealing, RC of Ni-Mn-Sn alloy first increases from 140 J.kg^−1^ for the as spun ribbons to 259.5 J.kg^−1^ and 269.4 J.kg^−1^ for 10 min and 60 min annealed samples, respectively, but decreases to 225.8 J.kg^−1^ for longer time 180 min annealed sample, which are listed in Table 1. Since the value of RC is calculated by the integration ΔS_M_ as a function of T within FWHM, the larger the ΔS_M_ and the wider FWHM are, the higher RC will be. 60 min annealed sample has a relative larger ΔS_M_, which is only a little bit lower than that of 10 min annealed sample, plus a relative wider FWHM. Therefore, it has the highest RC.

Additionally, magnetic hysteresis loss ΔM_hys_ must be considered for evaluating the usefulness of a magnetic refrigerant during a thermodynamic cycle since a high effective RC is favorable for its application. The value of the maximum magnetic hysteresis loss ΔM_hys_ for each sample, which is the dashed area shown in Fig. 2, is 57.1 J.kg^−1^, 185.5 J.kg^−1^, 168.6 J.kg^−1^ and 123.3 J.kg^−1^ for as spun, 10 min, 60 min, 180 min annealed samples, respectively, which is listed in Table 1. Magnetic hysteresis loss ΔM_hys_ is an intrinsic behavior for a first order transition. Generally, the more obvious and violent the first order transition is, the larger the magnetic hysteresis is. For the as spun ribbons, due to its inhomogeneous composition and low atomic order degree, the martensitic transition is mild since it undergoes a longer temperature range ΔT_A-M_, therefore, it has the lowest magnetic hysteresis loss ΔM_hys_. After a short time (10 min) annealing on this as spun ribbons, L2_1_ phase rapidly becomes homogeneous and uniform, leading to an obvious and violent martensitic transition. Additionally, the stress formed during rapid solidification has no time to release in a short time, resulting in a very large magnetic hysteresis ΔM_hys_ of 185.5 J.kg^−1^. As the increase of annealing time, the stress can gradually release and at the same time, the grain growth can also reduce frictions^25^,^26^, which will further lead to a lower hysteresis. Therefore, magnetic hysteresis for 60 min sample reduces to 168.6 J.Kg^−1^ and further reduces to 123.3 J.Kg^−1^ for 180 min annealed sample with the prolonging of annealing time.

For rapid quenched sample like melt-spun ribbons without annealing, the stress and defects formed during rapid quench will be reduced after annealing for a period of time, and further annealing will have no much influence. 60 min annealing should be enough to stress release and defect elimination, further increase of annealing time will have no much improvement. At the same time, overtime annealing will deteriorate the homogeneity of the composition as shown in Table 2, which will increase ΔM_hys_. While, as listed in Table 1, ΔM_hys_ for ribbons annealed for 180 min, still dramatically reduced about 45 J.kg^−1^ compared with that of annealed for 60 min. There should have other reasons for this decrease of ΔM_hys_ for overtime annealing. Except for the stress formed during solidification process, other kinds of stress developed during the reversible martensitic transformation, such as those caused by a large degree of grain constraints, affect the transformation range, which also can cause a large magnetic hysteresis^27^. In conventional shape memory alloys, elastic energy storage has been linked to grain size to thickness ratio^28^,^29^ during superelastic loading and, thus, thermally or magnetically induced martensitic transformation ranges should also be affected by this microstructural parameter. Samples with large grain to thickness ratios require less thermal or mechanical (or magnetic) driving force to achieve the same response than samples with small grain to thickness ratios.

**Figure 4:**
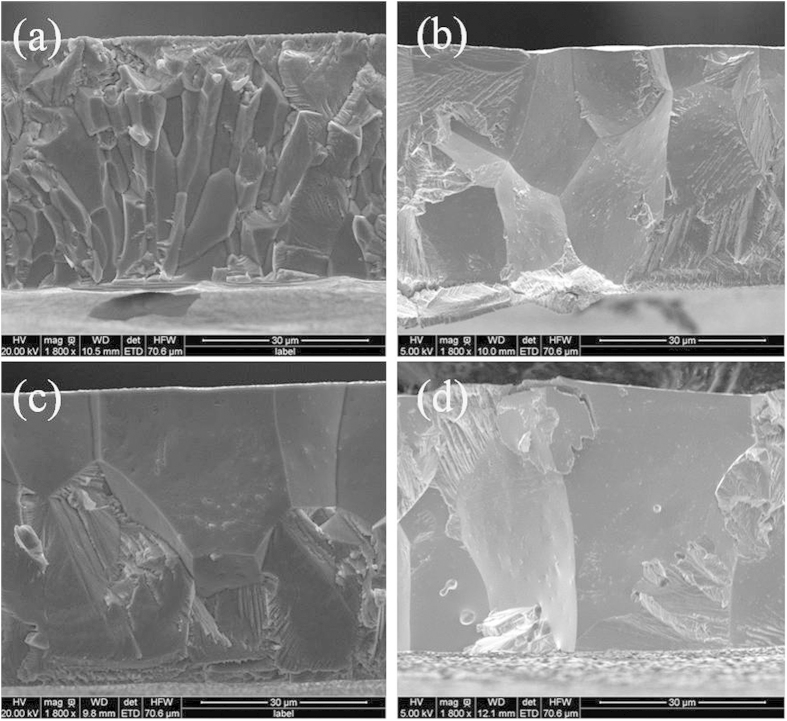
SEM image of fracture surface for (**a**) as spun ribbon, (**b**) 10 min, (**c**) 60 min and (**d**) 180 min annealed ribbon.

In order to find out how the grain size affects magnetic hysteresis, SEM observation on fracture surface of the ribbons was carried out for all samples. As shown in Fig. 4, all the ribbons have a similar thickness of about 50 μm. It can be seen that the grain size also increases with the increasing of annealing time. Figure 4a shows that the as-spun ribbon consists of thin columnar crystals with diameters of about 4 μm. Columnar crystals proceed with a lateral growth to about 13 μm after 10 min annealing. Meanwhile, the grains have three or four layers across the section as shown in Fig. 4b. Then the columnar crystals further grow to an average of 20 μm after annealed 60 min, at the same time, crystals continue its longitudinal growth, thus the grains have only two layers which can be seen in Fig. 4c. Then they finally grow into an average diameter of 26 μm after annealing for 180 min, when the whole ribbon is made up with one grain/one layer running through the cross section as shown in Fig. 4d. According to SEM results, for the annealed ribbons, the size of grains has a great enhancement along with the increase of annealing time. Grain constraints and elastic energy storage is related to gain sizes, which can explain the decrease of hysteresis upon annealing very well. Therefore, minimizing stored elastic energy and strain/stress is beneficial to reduce magnetic hysteresis. And one possible method to reduce magnetic hysteresis is to have gain size as large as possible, for example single crystal materials may have the lowest hysteresis^25^.

Returning to RC, the effective cooling capacity RC_eff_ is defined as a more reasonable criterion for assessing the cooling efficiency. RC_eff_ can be obtained by subtracting the average magnetic hysteresis loss during martensitic transition from RC value. So the values of RC_eff_ for all the studied samples are 65.9 J.kg^−1^, 102.2 J.kg^−1^, 115.4 J.kg^−1^ and 100.9 J.kg^−1^, respectively as listed in Table.1. It’s obvious that short time (10 min) heat treatment dramatically increase the ribbons’ RC_eff_ from 65.9 to 102.2 J.kg^−1^, which is mostly due to the large increase of ΔS_M_. And after a longer annealing (60 min) time, RC_eff_ still increase to 115.4 J.kg^−1^, which may due to the wider temperature range of ΔS_M_ since ΔS_M_ has a little bit decrease. However, with further increasing of annealing time (180 min), its RC_eff_ decreases to 100.9 J.kg^−1^. For RC_eff_, the longer the annealing time will lead to the lower the magnetic hysteresis, but ΔS_M_ also decrease substantially; therefore, the highest RC_eff_ appears when the decrease of magnetic hysteresis can’t compensate for the deterioration of the entropy change. Only after a proper time of annealing, highest RC_eff_ will be obtained; in our case, 60 min annealing is the optimal condition. The RC_eff_ of ribbons annealed for 60 min increased almost twice compared with the unannealed (as spun) ribbons.

### Atomic Ordering Degree and Magnetic Refrigeration Performance

As discussed above, atomic ordering degree AOD has an important effect on thermal hysteresis, ΔT_hys_, magnetic hysteresis ΔM_hys_, magnetization difference between austenitic phase and martensitic phase ΔM_A-M_ and magnetic entropy change ΔS_M_. In this section, atomic ordering degree will be investigated.

It has been mentioned above that in Ni-Mn-Sn alloys, there exists an ordering transition from relatively disordered cubic B_2_ phase to long-range ordered L2_1_ phase, as solidified from the high temperature melt^30^,^31^. As for L2_1_ phase, Mn and Sn atoms occupy their corresponding sites, respectively. Whereas in the high-temperature B2 phase, they are being evenly distributed. The B2-L2_1_ transition occurs at a certain temperature above 800 K depending on its composition. The atomic ordering of L2_1_ phase is also strongly influenced by annealing time. As discussed above, the magnetization change between austenitic phase and martensitic phase, and the enhanced magnetic entropy change ΔS_M_ upon annealing was attributed to the increase of atomic ordering degree. Generally, the higher the atom ordering degree is, the higher magnetization difference across martensitic transition has, and the better the magnetocaloric effect has.

**Figure 5:**
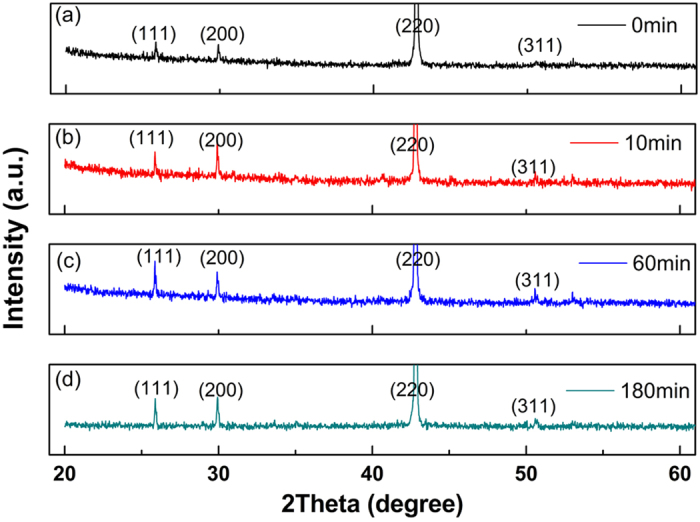
XRD patterns for ribbons annealed for (**a**) 0 min, (**b**) 10 min, (**c**) 60 min and (**d**) 180 min annealed ribbon.

In order to confirm the influence of annealing on the atomic ordering degree, and then prove its effect on magnetic properties, XRD was carried out on all the samples as shown in Fig. 5, in which all the peaks is corresponding to Heusler cubic L2_1_ structure, which indicates that all of the samples are austenitic phase. No martensitic phase was observed since martensite transition temperatures T_M_ are below room temperature. B2 phase is a high-temperature phase compare to L2_1_ phase. This B2-L2_1_ transition takes place promptly even less than 0.5 s in the process of cooling^32^. So even quenching from B2 phase range, all samples are indexed to be L2_1_ phase no matter annealed or unannealed. That is the reason why the as spun ribbons present L2_1_ phase. But because of the very high cooling rate (more than 10^5^ K/s), the as spun ribbon still reserve some disordered state. Post-heat treatment can realize the structural relaxation and the grain growth, thus gradually eliminating the disordered state through atomic diffusion.

Annealing would produce a great influence on the atomic order degree. The L2_1_ order degree can be qualitatively evaluated from the evolution of the intensities of superlattice reflection peaks, such as (111) and (311), whereas the evolution of the (111) peak appears most strikingly^33^,^34^. In the literatures^33^,^34^,^35^, the evolution of the order parameter associated with the L2_1_ structure was monitored based on 
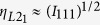
. Figure 5 shows the XRD patterns of the four samples. In order to rule out the measuring error, here AOD (atomic ordering degree) was defined as I(111)/I(220), where (220) is the strongest diffraction peak of L2_1_ phase. AOD is 0.06, 0.09, 0.11 and 0.07 for as-spun ribbons, 10 min, 60 min and 180 min annealing ribbons respectively, which is listed in Table 1. One can find that quenching directly from the melt gives a low AOD of 0.06, which gradually increases to 0.09 and 0.11, then decreases to 0.07 when annealing from 0 min to 180 min. With proper annealing time, AOD is improved; therefore ΔM_A-M_, ΔS_M_, and ΔT_hys_ ΔT_A-M_ have been improved greatly compared with unannealed ribbons as shown in Table 1. Even for the overtime annealed (180 min) samples, it still has higher ΔS_M_ and RC_eff_ than that of unannealed sample.

It is well known, atomic ordering degree AOD depends on microstructure and degree of crystallinity. Generally, longer annealing time is beneficial to obtain a higher AOD. In fact, the AOD for 60 min annealed sample is highest, but its ΔS_M_ is not the largest. While, 10 min annealed sample with a mild AOD but has the highest ΔS_M_. For excessive longer annealing time for example 180 mins, its AOD decreases. Therefore, except for annealing time, other factors, such as composition may become a more important issue to influence AOD and then affects magnetic properties.

### Composition evolution and magnetic entropy change

From the above results and discussions, annealing time favors to achieve a higher AOD and then to have higher magnetic refrigeration performance, such as higher ΔM_A-M_, ΔS_M_, and RC_eff_, lower ΔM_hys_ and ΔT_A-M_. However, over time annealing, for example 180 min, AOD will decrease from 0.11 to 0.07, therefore a decreasing of ΔS_M_ from ~40 Jkg^−1^K^−1^ to ~30 Jkg^−1^K^−1^, and increasing of ΔT_A-M_ from 3 K to 4 K. What is the under reason for the decrease of atomic ordering degree AOD becomes an important question. Furthermore, AOD value of 60 min is higher than that of 10 min, but the latter has the highest ΔS_M_ while it has similar ΔM_A-M_. There are some previous researches^36^,^37^,^38^ which ascribed the decreasing of atom ordering degree for ribbons with over time annealing to the variations of vacancy concentration, but no evidence was provided so far.

**Figure 6:**
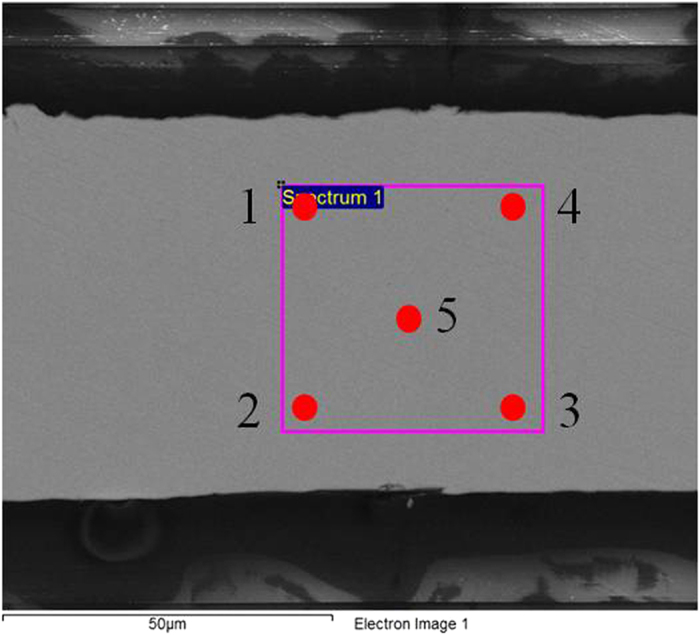
SEM image for 10 min annealed ribbon to give an example of how composition deviation was measured and calculated.

In our research, it is assumed except for AOD, that composition evolution may also play an important role. Here, a composition deviation ζ is introduced to investigate composition evolution upon annealing. Figure 6 is SEM image for one ribbon annealed for 10 min, which shows the points of EDS has been done. The average composition for each component in Ni-Mn-Sn as spun sample is defined as Ni_S0_, Mn_S0_ and Sn_S0_; the composition of the five points in the rectangle area is defined as Ni_S1_, Mn_S1_ and Sn_S1_ to Ni_S5_, Mn_S5_ and Sn_S5_. So the composition deviation for Ni can be got from:


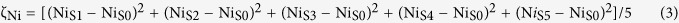


Then ζ_Mn_ and ζ_Sn_ can be got in the same way. Thus the composition deviation ζ for all the components in a sample is calculated by:


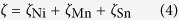


In order to minimize measurement errors, three different areas a, b and c were selected randomly in 10 min and 180 min annealed ribbons. All rectangles are in the same size and keep a same distance from the roll-touched surface. According to the method described above, an average composition deviation ζ is obtained after measured at least three different areas a, b and c by EDS, which is shown in Table 2. It is obvious that ζ for 180 min annealed ribbons is all larger than that of 10 min annealed ribbons, which indicates that the former is more inhomogeneous than the latter. This result proves that the composition also change along with atomic ordering degree upon annealing. Atomic ordering degree AOD is enhanced by increase of annealing time, but the composition deviation ζ is also enlarged. Therefore, there is a equilibrium point, when the increase of AOD can not be compensated by the enlarge of ζ. Thus an optimal annealing time is needed to achieve high magnetic refrigeration properties.

## Conclusions

The magnetic refrigeration properties, such as ΔT_A-M_, ΔM_hys_, ΔS_M_, and RC_eff_, of melt-spun Ni_43_Mn_46_Sn_11_ ribbons after different annealing time have been systematically investigated. An extremely large magnetic entropy change ΔS_M_ of 43.2 J kg^−1^K^−1^ in a field change of 5 T was achieved only after short time annealing (10 min) of the as-spun ribbons, but it gradually decreases to 32.3 J kg^−1^K^−1^ as annealing time increases to 180 min. At the same time, 10 min annealed ribbons have the largest ΔM_hys_, while ΔM_hys_ decreases with the increasing of annealing time but with a compensation of decrease of ΔS_M_. As a result, the largest RC_eff_ of 115.4 J.kg^−1^ was appeared in the 60 min annealed ribbons. Annealing also favors to have a lower thermal hysteresis, but over time annealing is also unfavorable. For samples with different annealing time, a maximum ΔS_M_ of 43.2 J.kg^−1^K^−1^, a lowest ΔM_hys_ of 123.3 J.kg^−1^, a maximum RC_eff_ of 115.4 J.kg^−1^ could be obtained in samples with different annealing time. While annealing time has no much influence on ΔT_A-M_ and ΔT_hys_. An optimal annealing time is 60 min that is it has not only a large magnetic entropy change of 41.4 J.kg^−1^K^−1^, but also a large effective cooling capacity of 115.4 J.kg^−1^. The ΔS_M_ increases for 125% and the RC_eff_ increases for 75% comparing the optimal annealing with unannealed ribbons.

Atomic ordering degree, microstructure, and composition have been applied to explain the change of magnetic refrigeration properties on different annealing time. Atomic ordering degree (AOD) enhancement by annealing attributed to a higer ΔM_A-M_, RC_eff_, ΔS_M_. Magnetic hysteresis loss ΔM_hys_ is also influenced by microstructure and composition. The larger crystal size and the uniform of the composition, the lower ΔM_hys_. Over time annealing leads to a large composition deviation, which is unfavorable to get an optimal magnetic refrigeration performance.

## Methods

The as-cast ingot of Ni_43_Mn_46_Sn_11_ was prepared by arc-melting under a high purity argon atmosphere. The purities of all raw elements are better than 99.9%. An additional 1wt. % Mn was added to compensate for its evaporation. The arc-melted ingot was cut into small pieces for use in the following melt-spun experiment. The small pieces were induction melted in a quartz tube with a rectangular nozzle (6 mm*0.5 mm) and then ejected onto a copper wheel rotating at a surface velocity of 15 m/s.

The melt-spun ribbons were sealed in quartz tubes, which were first evacuated, then filled with argon gas, and after that they were annealed at 1173 K for different time. After annealing, the quartz tube was quenched by water. The annealing time varied from 0 to 180 min.

The crystal structure analyses were carried out by x-ray diffraction (XRD) using Cu-K_**α**_ radiation at room temperature. The microstructure and composition were determined by scanning electron microscopy (SEM) using a JSM–6700 F microscope equipped with energy dispersive spectrometry (EDS). The magnetic measurements were carried out using a super conducting quantum interference device (SQUID).

## Additional Information

**How to cite this article**: Zhang, Y. *et al*. Enhanced magnetic refrigeration properties in Mn-rich Ni-Mn-Sn ribbons by optimal annealing. *Sci. Rep*. **5**, 11010; doi: 10.1038/srep11010 (2015).

## Figures and Tables

**Table 1:** Physical properties of ribbons annealed for different time.

	**0 min**	**10 min**	**60 min**	**180 min**
**A_s_**	211	219 K	220 K	221 K
**ΔT_A-M_**	11K	3 K	3.5 K	4 K
**ΔT_hys_**	10 K	7 K	7 K	7 K
**M_A_**	65 emu/g	75 emu/g	75 emu/g	67 emu/g
**M_M_**	35 emu/g	20 emu/g	20 emu/g	20 emu/g
**ΔM_A-M_**	30 emu/g	55 emu/g	55 emu/g	47 emu/g
**ΔM_hys_**	57.1 J.kg^−1^	185.5 J.kg^−1^	168.6 J.kg^−1^	123.3 J.kg^−1^
**ΔS_M_**	18.4 J kg^−1^K^−1^	43.2 J.kg^−1^K^−1^	41.4 J.kg^−1^K^−1^	32.3 J.kg^−1^K^−1^
**RC**	107.0 J.kg^−1^	201.0 J.kg^−1^	221.0 J.kg^−1^	185.0 J.kg^−1^
**RC**_**eff**_	65.9 J.kg^−1^	102.2 J.kg^−1^	115.4 J.kg^−1^	100.9 J.kg^−1^
**AOD**	0.06	0.09	0.11	0.07

A_s_: The starting temperature of austenitic phase;

ΔT_A-M_: Temperature range of martensitic phase transition;

ΔT_hys_: Thermal hysteresis;

M_A_: Magnetization of austenitic phase;

M_M_: Magnetization of martensitic phase;

ΔM_A-M_: Magnetization difference between austenitic phase and martensitic phase;

ΔM_hys_: Magnetic hysteresis loss;

ΔS_M_: Magnetic entropy change;

RC: refrigeration capacity, RC_eff_: effective cooling capacity;

AOD: Atomic ordering degree.

**Table 2:** Average composition deviation and three different areas’ composition diviation for 10 min and 180 min annealed ribbons.

	**ζ**	**ζ_a_**	**ζ_b_**	**ζ_c_**
**10 min**	0.086	0.113	0.062	0.084
**180 min**	0.224	0.235	0.249	0.189

## References

[b1] Pecharsky V. K. & Gschneidner K. A. Jr Giant magnetocaloric effect in Gd5(Si2Ge2). Phys. Rev. Lett. 78, 4494 (1997). 10.1103/PhysRevLett.84.4617 10990754

[b2] Hu F.-x. . Influence of negative lattice expansion and metamagnetic transition on magnetic entropy change in the compound LaFe11.4Si1.6. Appl. Phys. Lett. 78, 3675–3677 (2001).

[b3] Fujita A., Fujieda S., Hasegawa Y. & Fukamichi K. Itinerant-electron metamagnetic transition and large magnetocaloric effects in La (FexSi1-x)13 compounds and their hydrides. Phys. Rev. B 67, 104416 (2003).

[b4] Tegus O., Bruck E., Buschow K. & De Boer F. Transition-metal-based magnetic refrigerants for room-temperature applications. Nature 415, 150–152 (2002). 11805828 10.1038/415150a

[b5] Krenke T. . Inverse magnetocaloric effect in ferromagnetic Ni-Mn-CSn alloys. Nature Mater. 4, 450–454 (2005). 15895096 10.1038/nmat1395

[b6] Wood M. & Potter W. General analysis of magnetic refrigeration and its optimization using a new concept: maximization of refrigerant capacity. Cryogenics 25, 667–683 (1985).

[b7] Gschneidner K. Jr, Pecharsky V., Pecharsky A. & Zimm C. in Mater. Sci. Forum. 315, 69–76 (1999).

[b8] Provenzano V., Shapiro A. J. & Shull R. D. Reduction of hysteresis losses in the magnetic refrigerant Gd5Ge2Si2 by the addition of iron. Nature 429, 853–857 (2004). 15215859 10.1038/nature02657

[b9] Du J. . Large magnetocaloric effect and enhanced magnetic refrigeration in ternary Gd-based bulk metallic glasses. J. Appl. Phys. 103, 023918, 10.1063/1.2836956 (2008).

[b10] Luo Q., Zhao D. Q., Pan M. X. & Wang W. H. Magnetocaloric effect in Gd-based bulk metallic glasses. Appl. Phys. Lett. 89, 081914, 10.1063/1.2338770 (2006).

[b11] Yuan F., Du J. & Shen B. Controllable spin-glass behavior and large magnetocaloric effect in Gd-Ni-Al bulk metallic glasses. Appl. Phys. Lett. 101, 032405 (2012).

[b12] Du J. . Magnetocaloric effect and magnetic-field-induced shape recovery effect at room temperature in ferromagnetic Heusler alloy Ni–Mn–Sb. J. Phys. D: Appl. Phys. 40, 5523–5526, 10.1088/0022-3727/40/18/001 (2007).

[b13] Buchelnikov V. & Sokolovskiy V. Magnetocaloric effect in Ni-Mn-X (X = Ga, In, Sn, Sb) Heusler alloys. The Physics of Metals and Metallography 112, 633–665 (2011).

[b14] Liu J., Gottschall T., Skokov K. P., Moore J. D. & Gutfleisch O. Giant magnetocaloric effect driven by structural transitions. Nature Mater. 11, 620–626 (2012). 22635044 10.1038/nmat3334

[b15] Gschneidner K. Jr & Pecharsky V. Magnetocaloric materials. Annual Rev. Mater. Sci. 30, 387–429 (2000).

[b16] Shen B., Sun J., Hu F., Zhang H. & Cheng Z. Recent progress in exploring magnetocaloric materials. Adv. Mater. 21, 4545–4564 (2009).

[b17] Xuan H. . Effect of annealing on the martensitic transformation and magnetocaloric effect in Ni 44.1 Mn 44.2 Sn 11.7 ribbons. Appl. Phys. Lett. 92, 242506-242506-242503 (2008).

[b18] Zheng H. . Martensitic transformation in rapidly solidified Heusler Ni49Mn39Sn12 ribbons. Acta Mater. 59, 5692–5699 (2011).

[b19] Aksoy S., Acet M., Deen P., Ma osa L. & Planes A. Magnetic correlations in martensitic Ni-Mn-based Heusler shape-memory alloys: Neutron polarization analysis. Phys. Rev. B 79, 212401 (2009).

[b20] Ghosh A. & Mandal K. Effect of structural disorder on the magnetocaloric properties of Ni-Mn-Sn alloy. Appl. Phys. Lett. 104, 031905 (2014).

[b21] Zhang Y. . Enhanced large magnetic entropy change and effective cooling capacity of Ni43Mn46Sn11 alloys by a rapid solidification method. Scripta Mater. 104, 41–44, 10.1016/j.scriptamat.2015.04.004 (2015).

[b22] Shamberger P. J. & Ohuchi F. Hysteresis of the martensitic phase transition in magnetocaloric-effect Ni-Mn-Sn alloys. Phys.Rev. B 79, 144407 (2009).

[b23] Zhang Y. . Large magnetic entropy change and enhanced mechanical properties of Ni–Mn–Sn–C alloys. Scripta Mater. 75, 26–29, 10.1016/j.scriptamat.2013.11.009 (2014).

[b24] Sokolovskiy V. . First-principles investigation of chemical and structural disorder in magnetic Ni2Mn1+ xSn1-x Heusler alloys. Phys. Rev. B 86, 134418 (2012).

[b25] Wang W.-H., Chen J.-L., Liu Z.-h., Wu G.-H. & Zhan W.-S. Thermal hysteresis and friction of phase boundary motion in ferromagnetic Ni 52 Mn 23 Ga 25 single crystals. Phys. Rev. B 65, 012416 (2001).

[b26] Liu Z. . The influence of heat treatment on the magnetic and phase transformation properties of quaternary Heusler alloy Ni50Mn8Fe17Ga25 ribbons. Scripta Mater. 51, 1011–1015 (2004).

[b27] Bruno N. M. . The effect of heat treatments on Ni 43 Mn 42 Co 4 Sn 11 meta-magnetic shape memory alloys for magnetic refrigeration. Acta Mater. 74, 66–84 (2014).

[b28] Dvorak I. & Hawbolt E. Transformational elasticity in a polycrystalline Cu-Zn-Sn alloy. Metall. Trans. A 6, 95–99 (1975).

[b29] Somerday M., Wert J. & Comstock R. Jr Effect of grain size on the observed pseudoelastic behavior of a Cu-Zn-Al shape memory alloy. Metall. Mater. Trans. A 28, 2335–2341 (1997).

[b30] Ayuela A., Enkovaara J., Ullakko K. & Nieminen R. Structural properties of magnetic Heusler alloys. J. Phys.: Condensed Matter 11, 2017 (1999).

[b31] Wachtel E., Henninger F. & Predel B. Constitution and magnetic properties of Ni-Mn-Sn alloys-solid and liquid state. J. Magn. Magn. Mater. 38, 305–315 (1983).

[b32] Overholser R. W., Wuttig M. & Neumann D. Chemical ordering in Ni-Mn-Ga Heusler alloys. Scripta materialia 40, 1095–1102 (1999).

[b33] Wu D. . Atomic ordering effect in Ni50Mn37Sn13 magnetocaloric ribbons. Mater. Sci. Eng. A 534, 568–572 (2012).

[b34] Liu J., Woodcock T. G., Scheerbaum N. & Gutfleisch O. Influence of annealing on magnetic field-induced structural transformation and magnetocaloric effect in Ni-Mn-In-Co ribbons. Acta Mater. 57, 4911–4920 (2009).

[b35] Planes A. . Neutron diffraction study of long-range atomic order in Cu-Zn-Al shape memory alloys. J. Phys.: Condensed Matter 4, 553 (1992).

[b36] Gonzalez-Legarreta L. . Annealing Influence on the Microstructure and Magnetic Properties of Ni-Mn-In Alloys Ribbons. Journal of superconductivity and novel magnetism 25, 2431–2436 (2012).

[b37] Sanchez-Alarcos V., Recarte V., Perez-Landazabal J. & Cuello G. Correlation between atomic order and the characteristics of the structural and magnetic transformations in Ni-Mn-Ga shape memory alloys. Acta Mater. 55, 3883–3889 (2007).

[b38] Santamarta R. . Effect of atomic order on the martensitic transformation of Ni-Fe-Ga alloys. Scripta Mater. 54, 1985–1989 (2006).

